# What if we just give everything away?

**DOI:** 10.7554/eLife.74981

**Published:** 2021-11-05

**Authors:** Luke D Lavis

**Affiliations:** 1 Janelia Research Campus, Howard Hughes Medical Institute Ashburn United States

**Keywords:** sparks of change, open chemistry, fluorescent dyes, research culture

## Abstract

A group leader decided that his lab would share the fluorescent dyes they create, for free and without authorship requirements. Nearly 12,000 aliquots later, he reveals what has happened since.

It was the email every tool-builder hopes for. *“I recently saw a talk mentioning the dyes your lab developed. Is it possible to get a sample?”.*

The problem? This was the fifth request of the week, and it was only Tuesday.

I’ve always been a fluorescent dye nerd, stumbling into this field with my first ‘real’ job as a technician for a biotech company. I get to use chemistry principles to design and build colorful molecules that help visualize cellular activity. And in the process, I work with a vibrant community of biologists who constantly push the frontier of what can be done with a microscope. To them, better dyes mean new types of experiments, and someone is always excited to try out the latest molecule.

I dedicated my lab at HHMI’s Janelia Research Campus to ushering the archaic field of dye chemistry into the modern era. When I received that email in 2015, we had just discovered how a simple chemical modification could make dyes brighter and last longer. As is often the case in our discipline, we patented the idea and planned to see if companies would license and commercialize the technology; in the meantime, we shared our initial batches of dyes within a small network of collaborators. But now the word was getting out, and email requests were picking up. And for the first time in my career, I felt I had the opportunity to change how the scientific community accessed these molecules.

**Figure 1:**
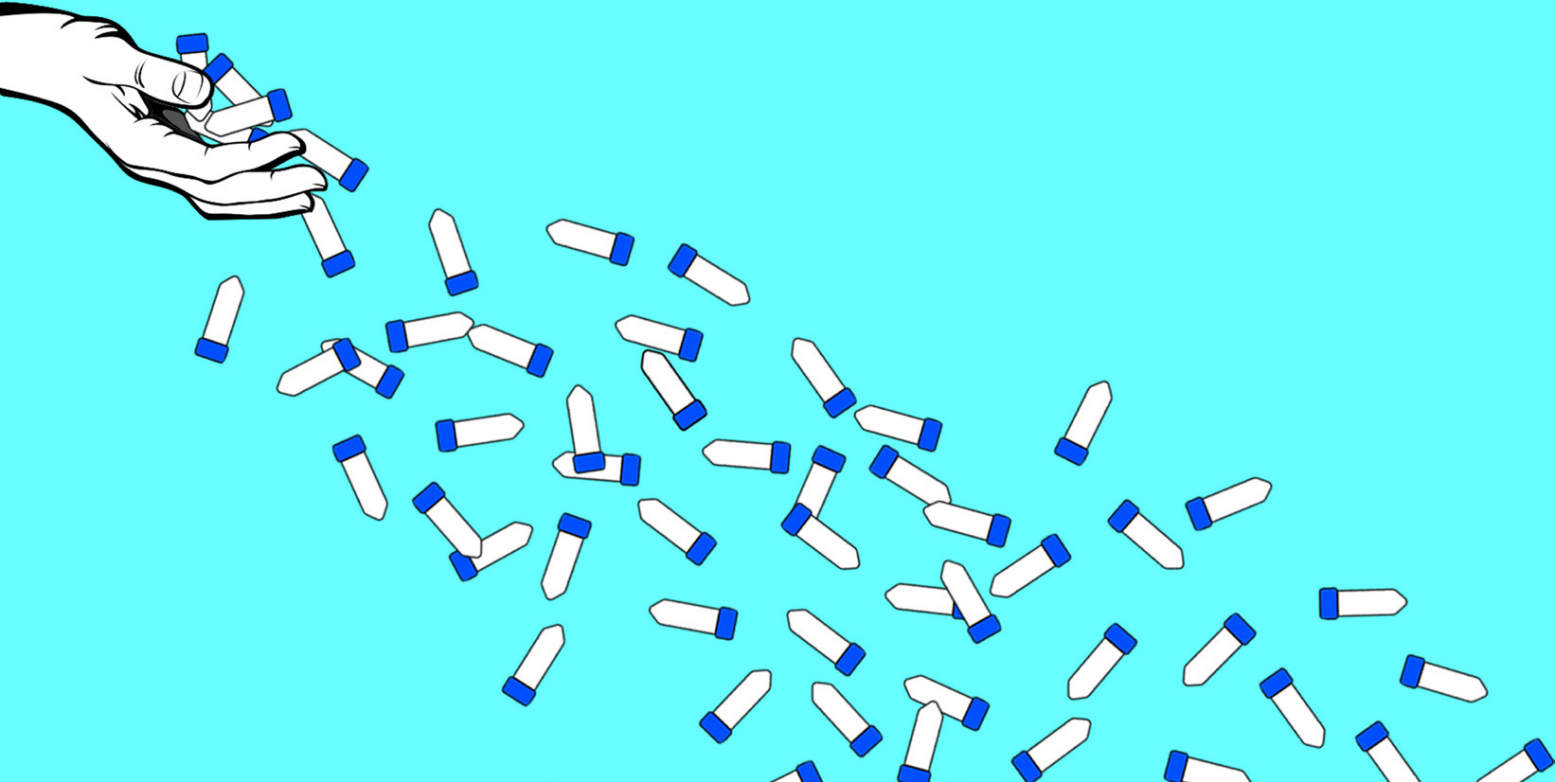
IMAGE CREDIT: Public domain.

Today, most scientists buy their dyes from biotech companies; product availability often depends on commercial factors such as market size or business model. Access to other molecules is limited to those who ‘know a chemist’ able to synthetize the dye and willing to share it, often in exchange for authorship. During my time in industry, I also learned that dyes are expensive to develop but often cheap to produce – leading to strikingly large profit margins, partly to recoup the research costs. In academia, however, development of new reagents is covered by governments or institutes. Chalk it up to my rural Oregon hippie upbringing, but I started to question why we couldn’t leverage this investment to broadly share fluorescent dyes.

I re-read the email, slipped on my Birkenstock clogs, and headed across the hall to talk to Jon Grimm, the senior scientist who developed our new dyes and a fellow industry expat. Standing in the chemistry lab, we debated whether we should wait for companies to license our molecules. This would be easier for us; we could move on to other projects and perhaps earn a few royalties to boot. But we could also miss an opportunity to impact the field just as the use of these reagents was taking off.

Eventually we dared to ask: what if we just give everything away*?* Share the dyes with anyone who gets in touch. No cost. No authorship requirements. Just cite our paper and pay the shipping if you can. A risky, but exciting no-holds-barred approach.

I knew we’d have backing from the top: 33 years earlier, my boss Gerry Rubin had shown up to a conference with 300 kits for making transgenic flies. Chemistry was also on our side. Because we had optimized the methods to synthetize the dyes, the price per aliquot was now down to mere pennies. So, I went back to my office and responded: “*Happy to help. Can you send me a shipping address?”.*

We didn’t formally advertise our decision, but over the following months our willingness to share became an open secret. We fielded more and more emails; Jon resynthesized material, prepared the aliquots, and I spent Sunday nights divvying them into little protective bags. In hindsight, we probably spent too long doing it this way, but I enjoyed directly interacting with all the scientists who put our tools to use.

After a few years our lab coordinator, Anastasia Osowski, helped us develop better ways to deal with the increasing number of messages. In 2019, we launched an online portal that allows researchers from nonprofit institutes to request dyes with a click of a mouse. Jon trained a string of talented chemistry technicians – Anand Muthusamy, Anthony Ayala, Natalie Falco, and Katie Holland – in our new synthetic methods. With them, our investment in optimizing dye chemistry paid off, and our project grew.

In the past four years, we have shared more than 50 types of dyes, packaged into 11,890 aliquots sent to over 500 labs in 32 countries. We have discovered the complexities of Australian customs and how much dry ice you need to ship to South Africa. It has been a fun ride – there is nothing like receiving feedback that says: “These dyes are so bright, I’m crying at the microscope”.

We’ve also learned a lot.

First, we can’t do everything – but neither can companies. We need to partner with industry to gain access to their distribution networks and economies of scale for some of our molecules, which are used by large numbers of scientists. But other ‘niche’ reagents simply do not have the market size to be viable products. This is where we step in.

Second, when dyes aren’t a limited resource, people try out-of-the-box ideas: labeling in whole animals, building new types of sensors, and even ways to ‘timestamp’ certain biological process as they unfold in the cell. Giving researchers the opportunity to exercise their creativity unfettered is one of the best ways to push science forward.

Third, not trading reagents for authorship frees up scientists – especially early in their careers – to do their best work without worrying about the byline. We still get credit in the reference list, but it is liberating to ‘talk science’ with graduate students and postdocs without authorship issues complicating the communication.

I feel privileged to work in an organization which recognizes the value of basic and applied research, but also has the resources and ambition to make tools available to all. My lab will continue to leverage the opportunities Janelia offers, and we’re expanding our ‘Open Chemistry’ initiative. We’re recruiting additional technicians who want to hone their synthesis skills and contribute to the community before heading off to graduate school. And we’re looking to partner with chemistry tool-builders who share our mindset at other institutions. Together, we can help drive the next biological discoveries by making reagents open and accessible.

## Share your experiences

This article is a Sparks of Change column, where people around the world share moments that illustrate how research culture is or should be changing. Have an interesting story to tell? See what we’re looking for and the best ways to get in touch here.

